# Supplementary material for: Diagnostic Value of the Vestibular Autorotation Test in Menière’s Disease, Vestibular Migraine and Menière’s Disease with Migraine
Source: Brain Sci. 2022 Oct 25;12(11):1432. doi: 10.3390/brainsci12111432 (PMC9688433; doi:10.3390/brainsci12111432)
Supplement: Supplementary file 1 [file brainsci-12-01432-s001.zip › brainsci-1965206-supplementary.pdf]

Supplementary Materials:

Table S1. Characteristics of VAT parameters in MD and VM patients.

Parameter	classification	MD (n = 69)	VM (n = 79)	
		n (%)	n (%)	p-value
Horizontal gain	decrease	21 (30.4)	5 (6.3)	<0.001***
	normal	45 (65.2)	34 (43.0)	0.01*
	increase	3 (4.3)	40 (50.6)	<0.001***
Horizontal phase	decrease	6 (8.7)	3 (3.8)	0.21
	normal	30 (43.5)	28 (35.4)	0.32
	increase	33 (47.8)	48 (60.8)	0.12
Vertical gain	decrease	6 (8.7)	4 (5.1)	0.58
	normal	60 (87.0)	73 (92.4)	0.27
	increase	3 (4.3)	2 (2.5)	0.54
Vertical phase	decrease	6 (8.7)	4 (5.1)	0.58
	normal	60 (87.0)	73 (92.4)	0.27
	increase	3 (4.3)	2 (2.5)	0.54
Asymmetry	normal	51 (73.9)	63 (79.7)	0.40
	abnormal	18 (26.1)	16 (20.3)	0.40

Note*：*$p < 0.05$, **$p < 0.01$, ***$p < 0.001$ Abbreviations: MD: Meniere's disease; VM: vestibular Migraine; VAT: vestibular autorotation test

Table S2. Identification of different parameters of VAT in VM and MD patients by multivariate binary logistic regression analysis.

Parameter	Classification	OR	95% CI	p-value
Horizontal gain	decrease	1.00	-	＜0.001***
	normal	0.28	0.09~0.87	0.03*
	increase	0.01	0.00~0.07	＜0.001***
Horizontal phase	decrease	1.00	-	0.99
	normal	0.96	0.15~6.32	0.97
	increase	1.03	0.16~6.45	0.98
Vertical gain	decrease	1.00	-	0.10
	normal	0.19	0.03~1.17	0.07
	increase	0.99	0.05~19.49	0.99
Vertical phase	decrease	1.00	-	0.10
	normal	0.19	0.03~1.17	0.07
	increase	0.99	0.05~19.49	0.99
Asymmetry	normal	1.00	-	1.00
	abnormal	1.08	0.39~2.99	0.88

Note**:** *$p < 0.05$, **$p < 0.01$, ***$p < 0.001$ ***Abbreviations***: MD, Meniere's disease; VM, vestibular migraine; OR, odds ratio; CI, confidence interval; VAT: vestibular autorotation test

Table S3. Cutoff value of different parameters of VAT by multivariate binary logistic regression analysis.

Test Result Variable(s)	Positive if Greater Than or Equal To[a]	Sensitivity	1 - Specificity
Horizontal gain	0.00	1.00	1.00
	0.32	0.96	0.49
	0.69	0.30	0.06
	1.00	0.00	0.00
Horizontal phase	0.00	1.00	1.00
	0.46	0.52	0.39
	0.59	0.09	0.04
	1.00	0.00	0.00
Vertical gain	0.00	1.00	1.00
	0.53	0.13	0.08
	0.60	0.09	0.05
	1.00	0.00	0.00
Vertical phase	0.00	1.00	1.00
	0.53	0.13	0.08
	0.60	0.09	0.05
	1.00	0.00	0.00
Asymmetry	0.00	1.00	1.00
	0.49	0.26	0.20
	1.00	0.00	0.00

Note:

The test result variable(s): Horizontal gain, Horizontal phase, Vertical gain, Vertical phase, Asymmetry has at least one tie between the positive actual state group and the negative actual state group.

a. The smallest cutoff value is the minimum observed test value minus 1, and the largest cutoff value is the maximum observed test value plus 1. All the other cutoff values are the averages of two consecutive ordered observed test values.

Table S4. VAT testing method.

Test principle	Subjects were asked to perform active head movements over a rising frequency, ranging from 2.0 to 6.0 Hz, which can detect a closer-to-real state of the vestibular function, and they were instructed to perform head rotations on horizontal and vertical planes. An algorithm was used for spectrum analysis to obtain gain, phase, and asymmetry.	
Patients	During the test, the patient was required to fix the eyes on a target and move the head simultaneously in response to the beeps of the computer speaker (beeps frequency from slow to fast: 0.5 Hz→6 Hz, amplitude of head movement: 20° → 5°). The test was performed three times on horizontal and vertical planes.	
Parameter	Description	Calculation formula
Gain	The ratio of eye to head speed	Gain = PSD (Eye velocity)/PSD (Head velocity)
Phase	The time when eye movement lags behind head movement	Phase = PSD(t)/ PSD(T) x 360°
Asymmetry	Symmetry of left and right eye velocity	Asymmetry (%) =100% [(PSD right eye velocity - PSD left eye velocity)]/[(PSD right eye velocity + PSD left eye velocity)]

Notes: t: The interval time between the eye peak and the head peak in the same direction as before, T：Time between two adjacent head peaks. PSD (Power Spectral Density): An algorithm for spectrum analysis, which used the Fast Fourier Transformation (FFT) of time-domain signals such as “Eye speed” and “Head speed”, was used to generate the desired amplitude information at different frequencies using a PSD algorithm.

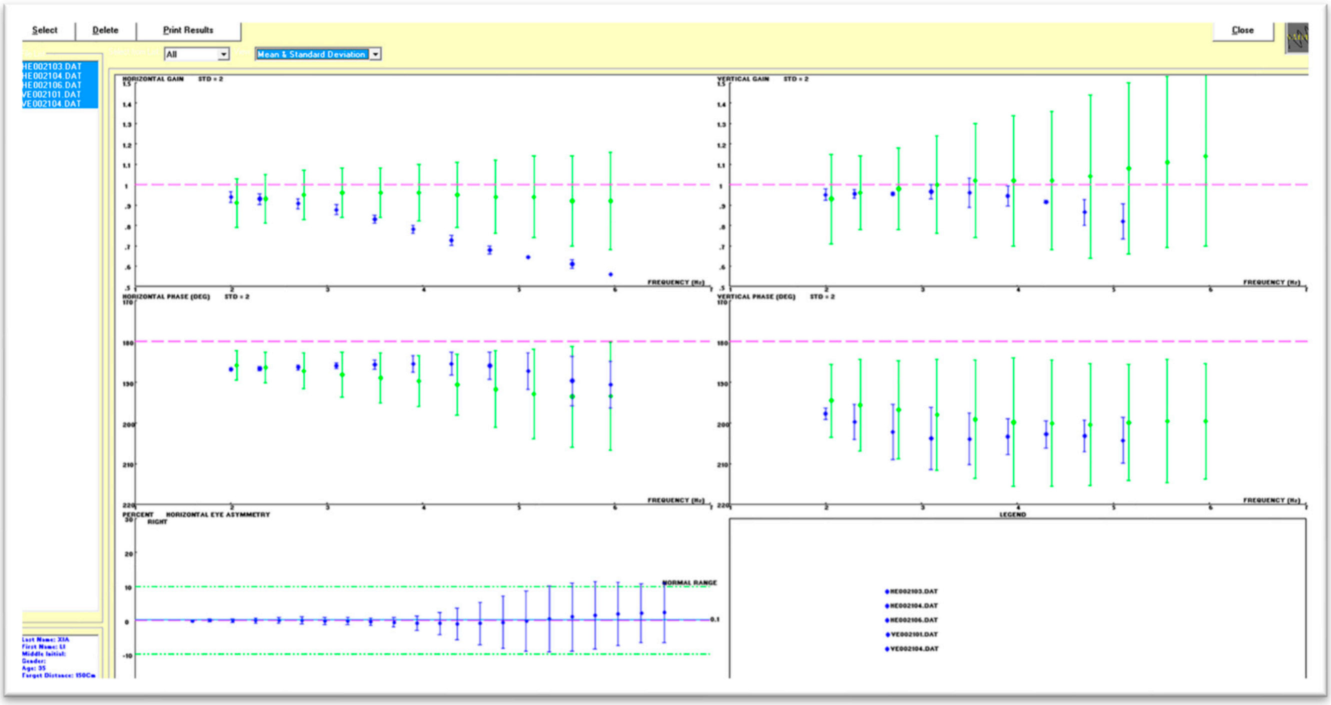

Figure S1. VAT result from a patient with MD (Meniere's disease). A decreased horizontal gain can be noted. a): Horizontal gain; b: Vertical gain; c: Horizontal phase; d: Vertical phase; e: Asymmetry

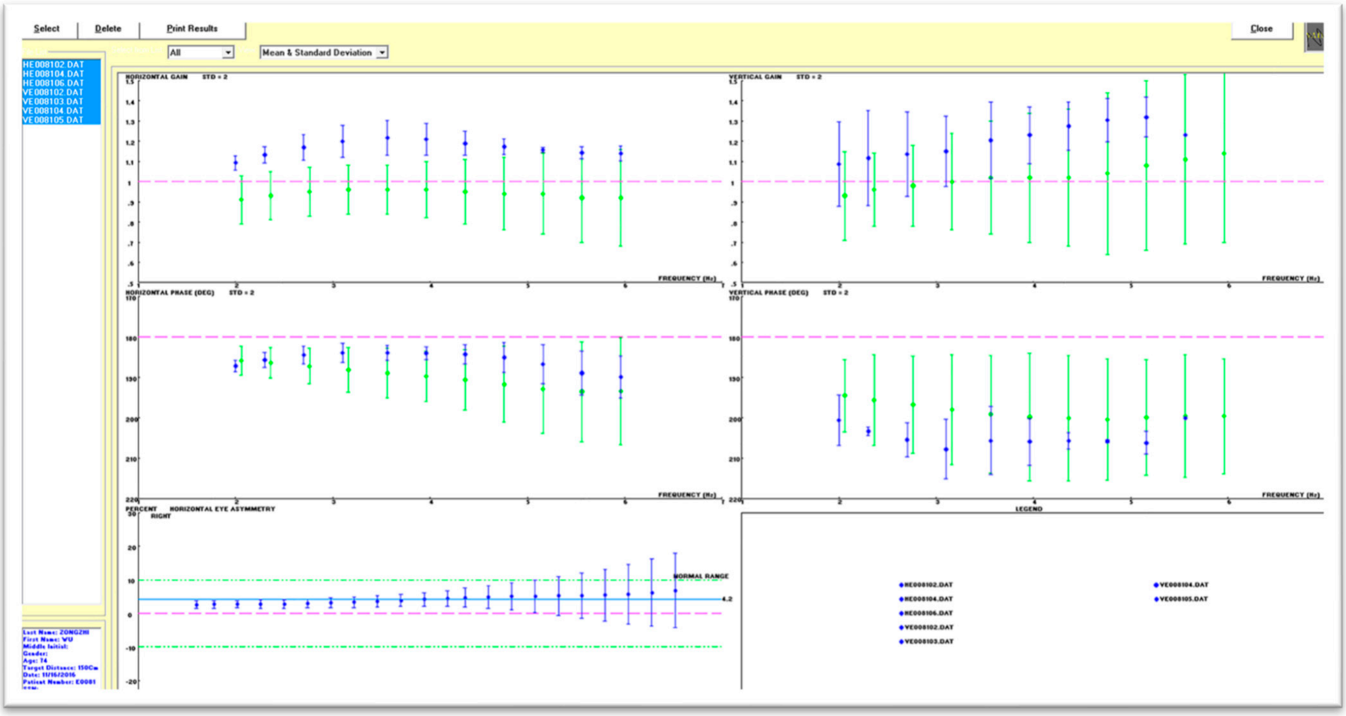

Figure. S2 VAT result from a patient with VM (vestibular migraine). An increased horizontal gain can be noted. a): Horizontal gain; b: Vertical gain; c: Horizontal phase; d: Vertical phase; e: Asymmetry
